# Cell type-dependent directional transcription at enhancers

**DOI:** 10.1093/nargab/lqaf007

**Published:** 2025-03-07

**Authors:** Saumya Agrawal, Emi Kanamaru, Yoriko Saito, Fumihiko Ishikawa, Michiel de Hoon

**Affiliations:** RIKEN Center for Integrative Medical Sciences, Yokohama 230-0045, Japan; RIKEN Center for Integrative Medical Sciences, Yokohama 230-0045, Japan; RIKEN Center for Integrative Medical Sciences, Yokohama 230-0045, Japan; RIKEN Center for Integrative Medical Sciences, Yokohama 230-0045, Japan; RIKEN Center for Integrative Medical Sciences, Yokohama 230-0045, Japan

## Abstract

Enhancers are noncoding regulatory regions in the genome that play essential roles in modulating gene expression. Previous work showed that enhancers are not transcriptionally silent but are characterized by bidirectional expression of short capped noncoding RNAs. Balanced bidirectional expression has therefore been used as a key feature for the detection of enhancers from transcriptome data. Instead, by analyzing FANTOM5 and other deep cap analysis gene expression transcriptome datasets, we find enhancer transcription preferentially in one direction in individual cell types. As the preferred direction of transcription of an enhancer can switch between cell types, balanced bidirectional enhancer expression may appear if transcriptome data are aggregated over cell types. 5′ single-cell RNA sequencing data showed that enhancers were almost exclusively expressed unidirectionally in a single cell. Reporter assay data demonstrated that the regulatory function of an enhancer does not depend on its preference for unidirectional or bidirectional expression. We conclude that requiring balanced bidirectional transcription for enhancer detection may discard most valid enhancers when applied to transcriptome data of a single cell type.

## Introduction

Enhancers are genomic regulatory elements that can affect expression of genes over megabases of genomic distances by chromatin looping [1, 2]. Enhancers produce capped transcripts known as enhancer RNAs (eRNAs) in a cell type-specific manner, with enhancer transcription associated with the regulatory activity of the enhancer [3, 4].

As eRNA transcription has been reported to occur bidirectionally [4], methods to identify enhancers using transcriptome data [5] commonly use balanced bidirectional transcription [e.g. in cap analysis gene expression (CAGE) [3, 6], PRO-cap [7], GRO-seq [8–10], or RNA-seq [11] data] as a signature feature of enhancers. However, the low abundance of eRNAs due to their rapid degradation by the exosome [12] complicates eRNA detection and hence an accurate assessment of enhancer expression directionality.

In this work, we analyzed recent very deep CAGE datasets [13–16], allowing the expression directionality of enhancers to be calculated accurately, and found that each enhancer typically has a preferred direction of expression in an individual cell type. We then developed a rigorous statistical model to analyze transcriptional directionality accurately for expression data with a lower sequencing depth, and found predominantly directional enhancer expression also in the previously analyzed data, with a cell type-dependent preferred direction of transcription. Our analysis suggests that bidirectional expression is not a necessary requirement to detect enhancers, especially for data obtained from a single cell type.

## Materials and methods

We define *p* as the probability for a transcriptome sequencing tag to be in the forward direction, and (1 – *p*) is the probability for a transcriptome sequencing tag to be in the reverse direction. Given this probability *p*, we use the binomial distribution to calculate the probability of *F* forward CAGE tags and *R* reverse CAGE tags for an enhancer with *F* + *R* CAGE tags in total:


\begin{equation*}{\mathrm{Pr}}\left( {F,R|p} \right) = \frac{{\left( {F + R} \right)!}}{{F!\;R!}}{p^F}{\left( {1 - p} \right)^R}.\end{equation*}

The directionality score, as defined previously [3, 4], quantifies the expression directionality of an enhancer in transcriptome sequencing data:


\begin{equation*}{\mathrm{directionality\;score}} = \;\frac{{F - R}}{{F + R}},\end{equation*}

where *F* and *R* are the number of sequence reads on the forward and reverse strands, respectively, of the enhancer. The directionality score ranges from −1 (for transcription in the reverse direction only) to +1 (for transcription in the forward direction only); the absolute value of the directionality score ranges from 0 for perfectly balanced bidirectional expression (i.e. equal levels of forward and reverse transcription) to +1 for strictly unidirectional expression. For an enhancer with probability *p*, the expected value of the directionality score is 2 *p* − 1.

**Figure 1.**
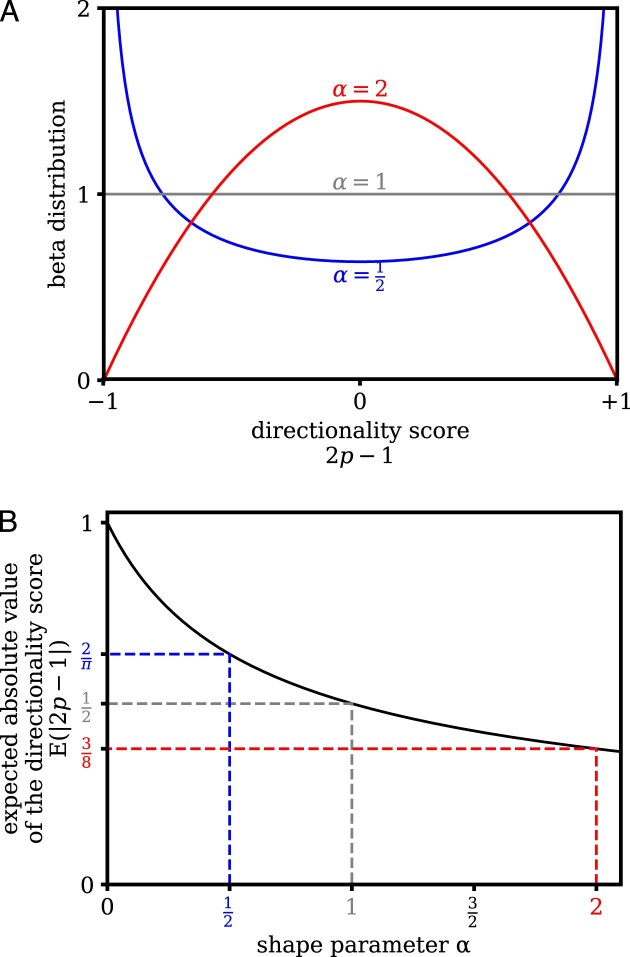
(**A**) The beta distribution with a shape parameter α < 1 (convex), α = 1 (uniform), and α > 1 (concave). (**B**) Expected absolute value of the directionality score, E(|2 *p* - 1|), as a function of the shape parameter α.

We model the distribution of *p* across enhancers using the beta distribution with the parameters *α* and *β* chosen equal, such that the distribution is symmetric around $p = \frac{1}{2}$:


\begin{equation*}{\mathrm{Pr}}\left( {p{\mathrm{|}}\alpha } \right) = \frac{1}{{{\ B}\left( {\alpha ,\alpha } \right)}}{p^{\alpha - 1}}{\left( {1 - p} \right)^{\alpha - 1}},\end{equation*}

where *B* is the beta function. The distribution is convex if *α* < 1, concave if *α* > 1, and uniform over its domain if *α* = 1 (Fig. 1A). The expected value of the absolute directionality score, |2 *p* - 1|, is a monotonically decreasing function (Fig. 1B):


\begin{equation*}{\rm E}\left( {\left| {2\;p\; - \;1} \right|} \right) = \frac{2}{{\alpha \;{\ B}\left( {\alpha ,\alpha } \right){4^\alpha }}}.\end{equation*}

For *α* = 0, the expected value of the absolute directionality score is 1, meaning that all enhancers are strictly unidirectionally transcribed. For *α* → ∞, the expected value of the absolute directionality score is 0, meaning that all enhancers are perfectly bidirectional, with an equal probability for forward and reverse transcription.

The compound distribution is a beta-binomial distribution:

${\mathrm{Pr}}( {F,R|\alpha } ) = \mathop \smallint \limits_0^1 {\mathrm{Pr}}( {F,R|p} ){\mathrm{Pr}}(p|\alpha ){\rm d}p = \frac{{( {F + R} )!}}{{F!\;R!}}\frac{{{\ B}( {F + \alpha ,R + \alpha } )}}{{{\ B}( {\alpha ,\alpha } )}}.$

Dropping constant terms, the log-likelihood function for *n* enhancers is

$L( \alpha ) = \mathop \sum \limits_{i = 1}^n {\mathrm{log}}( {\frac{{{\ B}( {{F_i} + \alpha ,{R_i} + \alpha } )}}{{{\ B}( {\alpha ,\alpha } )}}} ).$

The shape parameter α is estimated using the maximum-likelihood method; its statistical significance is calculated using the likelihood-ratio test by comparing max*_α_ L*(*α*) to *L*(*α*=1). Note that terms with *F* + *R* = 0 (enhancers with zero CAGE tags) do not contribute to the sum, while terms with *F* + *R* = 1 (enhancers with exactly one CAGE tag) do not depend on α and therefore also do not contribute to the estimation of the shape parameter.

## Results

**Figure 2.**
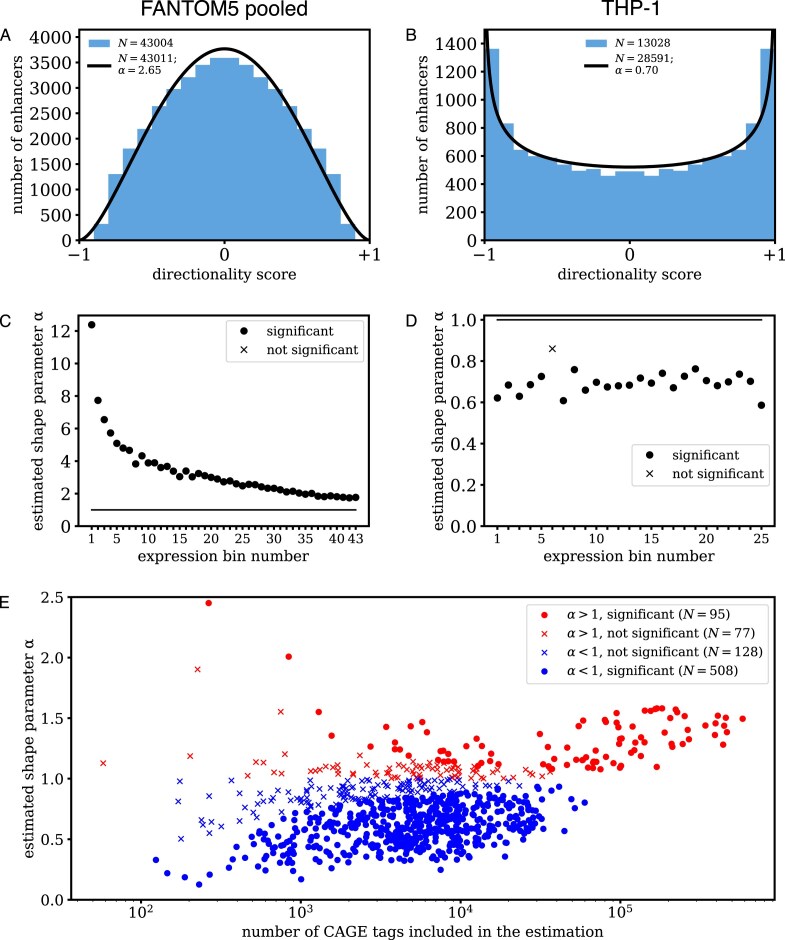
Distribution of the directionality score in (**A**) FANTOM5 pooled data [17] and in (**B**) THP-1 [13]. The estimated beta distribution is drawn as a curve; the histogram was calculated from enhancers with at least 10 CAGE tags. (**C**) Estimated shape parameter *α* in the FANTOM5 pooled data, split into 43 bins of increasing expression, each consisting of 1000 enhancers. (**D**) Estimated shape parameter *α* in the THP-1 data, split into 25 bins of increasing expression, each consisting of 1000 enhancers. (**E**) Scatter plot of the estimated shape parameter *α* versus the number of CAGE tags used in the estimation (Supplementary Table S1). Samples for which the estimated shape parameter *α* was significantly different from 1 are indicated by dots; samples for which the estimated shape parameter *α* was not significantly different from 1 are indicated by crosses. The number of samples in each category is shown in the legend.

We calculated the directionality score for the FANTOM5 Phase 1 enhancer set [3] using FANTOM5 Phase 1 CAGE data pooled over 808 samples (421 from primary cell types, 252 from cell lines, and 135 from tissues) [17]. Consistent with previous results [3], we found a concave distribution of the directionality score across enhancers (Fig. 2A). Enhancers with a zero directionality score were most common, and the prevalence of enhancers decreased as their absolute directionality score increased. In contrast, we found a convex distribution when using deep-sequenced CAGE data from the THP-1 leukemia cell line [13] (Fig. 2B), from human primary dermal fibroblast cells [14] (Supplementary Fig. S1A), from human iPS cells [15] (Supplementary Fig. S1B), and from acute myeloid leukemia (AML) primary cell samples [16] (Supplementary Fig. S1C). For a convex distribution, the prevalence of enhancers increases as their absolute directionality score increases, with strictly unidirectionally expressed enhancers being the most common.

Both intragenic and intergenic enhancers showed a concave distribution for the FANTOM5 Phase 1 pooled CAGE data (Supplementary Fig. S2A) and a convex distribution for the other CAGE datasets (Supplementary Fig. S2B–E). A convex distribution was also found for elongating transcripts (using GRO-seq data [18], PRO-seq data [7, 19], and NET-CAGE data [20]) and for transcription initiation (using GRO-cap data [19] and PRO-cap data [7]) in various cell lines (Supplementary Fig. S3).

As only the pooled FANTOM5 CAGE data showed a concave distribution (representing preferentially bidirectional enhancer expression), we asked whether the distribution of the directionality score would be concave or convex in each of the FANTOM5 samples separately. However, in each sample few enhancers had sufficient CAGE tags to estimate the directionality score accurately, precluding a direct calculation of a histogram. Instead, we model the shape of the directionality score distribution using a symmetric beta distribution (Fig. 1A), which depends on a single shape parameter α. This distribution is concave for α > 1, convex for α < 1, and uniform for α = 1. For α = 0, each enhancer has an absolute directionality of 1, with strictly unidirectional expression in the forward or reverse direction only. For α → ∞, all enhancers have an absolute directionality of 0, with an equal probability for forward or reverse expression. As the expected value of the absolute directionality score decreases monotonically as a function of the shape parameter α (Fig. 1B; see the ‘‘Materials and methods’’ section for details), we can use α as a measure for the preference of the population of enhancers toward unidirectional or bidirectional expression.

To model the number of forward and reverse CAGE tags at an enhancer given its probability *p* of expression in the forward direction, we use the binomial distribution. Compounding the beta and the binomial distribution yields the beta-binomial distribution to represent the number of forward and reverse CAGE tags at an enhancer. We then estimate the shape parameter α by fitting the beta-binomial distribution to the observed number of forward and reverse CAGE tags at each enhancer (see the ‘‘Materials and methods’’ section for details).

Fitting the beta-binomial distribution to the FANTOM5 pooled data gave a concave distribution (*P* < 10^−100^, likelihood ratio test), consistent with balanced bidirectional enhancer expression (Fig. 2A). Lowly expressed enhancers were more bidirectional than highly expressed enhancers, though the distribution tended toward bidirectional transcription in all expression bins (Fig. 2C). Next, we applied the beta-binomial model to each of the 808 samples in the FANTOM5 Phase 1 CAGE data set separately. The distribution was significantly (*P* < .05; likelihood ratio test) convex and concave for 508 and 95 samples, respectively; for the remaining 205, the distribution was neither significantly convex nor significantly concave (Fig. 2E). We conclude that enhancer expression is predominantly directional in the FANTOM5 Phase 1 samples analyzed separately, while balanced bidirectional enhancer expression is less common.

The estimated beta-binomial distribution was convex also for THP-1 (Fig. 2B; *P* < 10^−100^), dermal fibroblast (Supplementary Fig. S1A; *P* < 10^−100^), iPS cells (Supplementary Fig. S1B; *P* = 2 × 10^−82^), and AML primary cells (*P* = .17; Supplementary Fig. S1C), as well as for GRO-seq, PRO-seq, and NET-CAGE sequencing data measuring transcription elongation, and GRO-cap and PRO-cap data measuring transcription initiation (Supplementary Fig. S3), consistent with the enhancer expression being preferentially in the forward or reverse direction. These results are in agreement with the calculated histograms (Fig. 2B and Supplementary Fig. S1 and S3). Lowly expressed enhancers were more directionally expressed than highly expressed enhancers in most datasets, though the distribution tended toward directional expression in all expression bins (Fig. 2D and Supplementary Fig. S4).

As the FANTOM5 pooled data set was an order of magnitude larger than the other datasets (Supplementary Table S1), we performed additional numerical analyses to assess if the data size affected the estimated directionality distribution. After downsampling the FANTOM5 pooled data to the mean size (4948682 CAGE tags; Supplementary Table S1) of a single FANTOM5 sample, we found a concave distribution (Supplementary Fig. S5; *P* = 6 × 10^−28^, likelihood ratio test), with the estimated value of the shape parameter *α* in agreement with the value found for the highest expression bin in the pooled data (Fig. 2C). Furthermore, fitting the beta-binomial model to simulated data showed that the shape parameter *α* could be estimated reliably with at least ∼1000 CAGE tags on included enhancers (Supplementary Fig. S6A and C). All datasets in this study fulfill this requirement (Supplementary Table S1), except for 86 of the 808 samples in FANTOM5 Phase 1 CAGE data (Fig. 2E). The simulations indicated that using an insufficient number of CAGE tags typically yields a nonsignificant result rather than a significant but incorrect value (Supplementary Fig. S6B and D).

**Figure 3.**
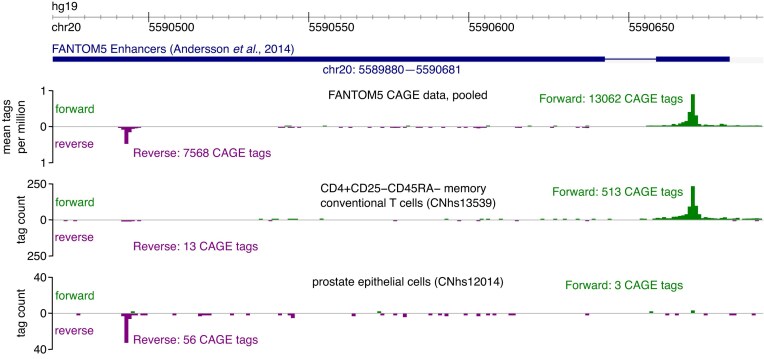
Genome browser view of the FANTOM5 enhancer on chr20 at locus 5589880–5590681 (human genome assembly hg19) with expression tracks for pooled data, for CD4^+^ CD25^−^ CD45RA^−^ memory conventional T cells, and for prostate epithelial cells. The number of CAGE tags in the forward and reverse directions is shown for each track. Transcription is predominantly in the forward direction in memory conventional T cells (directionality score = +0.95) and in the reverse direction in prostate epithelial cells (directionality score = −0.90).

Next, we investigated why enhancer expression was preferentially bidirectional in the pooled FANTOM5 CAGE but preferentially directional in the individual FANTOM5 samples. Because of the triangle inequality, the absolute directionality score in the pooled data is less than or equal to the absolute directionality score averaged over the samples, with equality achieved only if the directionality score has the same sign in all samples. Directionality scores of different signs may be found for a given enhancer due to sampling noise or due to genuine differences in the preferred direction of transcription. As an example of the latter, Fig. 3 shows an enhancer with bidirectional expression in pooled data, but with transcription mainly in the forward direction in memory conventional T cells and mainly in the reverse direction in prostate epithelial cells. The observed difference in the sign of the directionality score cannot be explained by sampling noise along (*P* = 1 × 10^−13^; binomial test), indicating that the preferred direction of transcription switches for this enhancer.

To assess if the directionality of an enhancer varied significantly across FANTOM5 samples, we performed the χ^2^-test for each enhancer across all samples for which the enhancer had at least 10 CAGE tags, requiring at least 2 such samples. We found that 9563 out of 21 664 enhancers (44.1%) had significantly (*P* < .05) varying directionality scores across samples. Of these, 3577 significantly changed their transcription direction in at least one sample (binomial test, *P* < .05; see Supplementary Methods for details) (Supplementary Table S2). In contrast, for the THP-1 time course, we found 1144 out of 4375 enhancers (26.1%) with a significantly changing directionality score across samples, with 188 enhancers (4.3%) significantly changing their transcription direction in at least one timepoint. As this is less than the significance threshold of 0.05, the enhancers with a significant *P*-value for transcription direction switching in the THP-1 time course may have been false positives.

**Figure 4.**
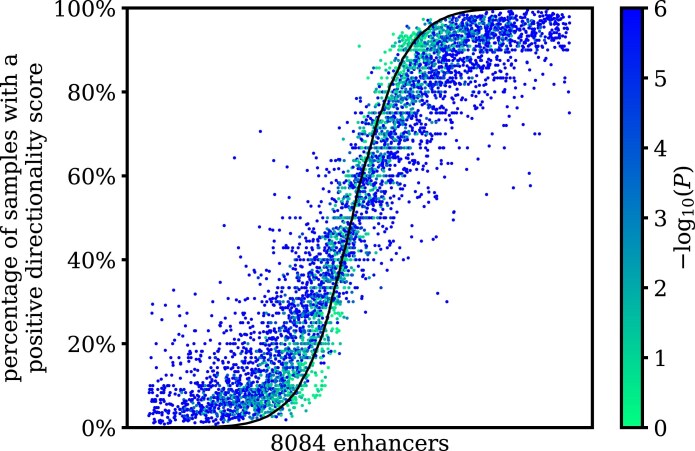
Percentage of samples with more transcription in the forward direction than in the reverse direction, for the 8084 enhancers in FANTOM5 CAGE data with at least 10 CAGE tags in at least 10 samples. Dots are colored by their statistical significance of the variation in the directionality score across samples for each enhancer. The black curve shows the expected percentage for each enhancer under the null hypothesis that its probability *p* of transcription in the forward direction is the same across samples. Enhancers are sorted by this expected percentage under the null hypothesis.

Next, we selected the 8084 enhancers that had at least 10 samples with at least 10 CAGE tags each in the FANTOM5 CAGE data, and counted how many of those samples had a positive or negative directionality score. Due to sampling noise, an enhancer may have some samples with a positive directionality score and some samples with a negative directionality score, even if its probability *p* of transcription in the forward direction does not vary across samples. Fig. 4 shows for each enhancer the number of samples with a positive directionality score as a percentage of the number of samples with a nonzero directionality score, together with the expected percentage under the background model. A lower absolute directionality score for pooled data is found for an enhancer with directionality scores of opposite signs in different samples, as they will cancel each other to some extent. For 5152 (63.7%) out of 8084 enhancers, the expected percentage under the background model had such a combination of directionality scores of opposite signs (Fig. 4). On average, the actual percentage was 5.2 percentage points closer to 50% than the expected percentage under the background model, resulting in more cancellation of positive and negative directionality scores when pooling. We concluded that both sampling noise and genuine switching of the transcription direction across samples contributed to the emergence of a preferentially bidirectional distribution of the directionality scores when pooling.

**Figure 5.**
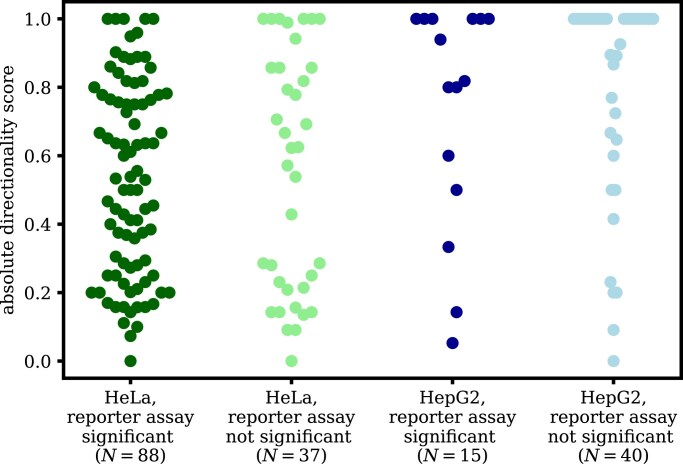
Enhancer expression directionality analysis of previously published reporter assay data [3]. Dots indicate the absolute directionality score in the FANTOM5 CAGE data calculated for the HeLa and HepG2 samples in the FANTOM5 Phase 1 set and are shown separately depending on the statistical significance of the measured reporter activities in each cell type.

We used previously published reporter assay data [3] to assess if the functionality of enhancers is associated with their directionality. We separated enhancers by the statistical significance of their reporter assay results obtained in the cervical carcinoma cell line HeLa and the human hepatocellular carcinoma cell line HepG2 cells, reported previously [3], and calculated the absolute directionality score using the FANTOM5 CAGE data for the HeLa and HepG2 samples, respectively. We found no statistically significant difference in the directionality scores between enhancers with significant and nonsignificant reporter activities in HeLa (Mann–Whitney test; *P* = .70, *N* = 125) and HepG2 (Mann–Whitney test; *P* = .98, *N* = 55) (Fig. 5).

**Figure 6.**
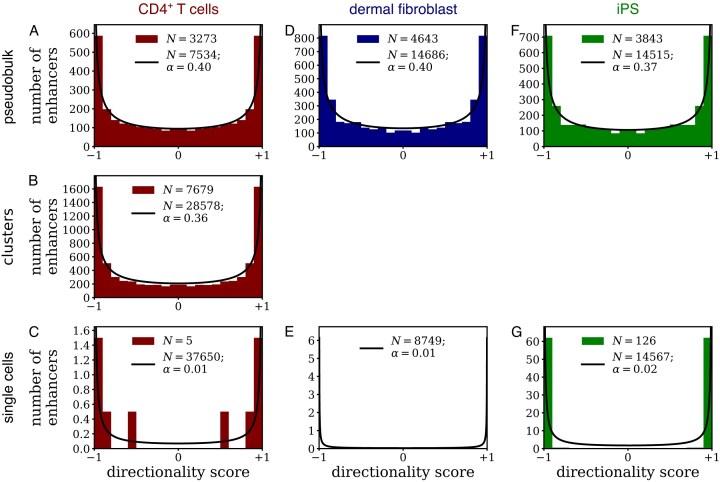
Distribution of the directionality score calculated from previously published 5′ single-cell RNA sequencing data in CD4^+^ T cells (**A**–**C**) [21], human primary dermal fibroblast cells (**D**, **E**) [22], and human iPS cells (**F**, **G**) [22]. Panels (A, D, and F) show the distribution for pseudobulk data generated by summing the CAGE data over single cells; panel (B) shows the distribution for clusters of CD4^+^ T cells [21]; and panels (C, E, and G) show the distribution for single cells.

An analysis of 5′ single-cell RNA sequencing of CD4^+^ T cells [21] revealed preferentially directional enhancer expression in the bulk data (α = 0.399; *P* < 10^−100^, likelihood ratio test; Fig. 6A), as expected for CAGE data for a single cell type. Preferentially directional enhancer expression (α = 0.357; *P* < 10^−100^, likelihood ratio test) was also found in the single-cell data separated into 15 UMAP (uniform manifold approximation and projection) clusters [21]; enhancer expression in these clusters was significantly (*P* = 2 × 10^−6^, likelihood ratio test) more directional than in the pseudobulk data (Fig. 6B). Bidirectional expression of an enhancer was even more rare in individual cells (α = 0.014; *P* < 10^−100^, likelihood ratio test; Fig. 6C). As a case in point, of 33 140 enhancers with exactly 2 CAGE tags, 32 714 had both CAGE tags pointing in the same direction, while only 426 had 1 forward and 1 reverse CAGE tag (*P* < 10^−100^, binomial test) (Supplementary Fig. S7). 5′ single-cell RNA sequencing of human primary dermal fibroblast and human iPS cells [22] similarly showed preferentially directional enhancer expression in bulk data and almost exclusively unidirectional enhancer expression in individual cells (dermal fibroblast: 233 out of 10 472; iPS cells: 109 out of 8158; both *P* < 10^−100^, binomial test; Fig. 6D–G and Supplementary Fig. S7). We note that these single-cell data do not distinguish between alleles, implying that any remaining bidirectional expression may be due to unidirectional expression in opposite directions in the two alleles.

Our analysis suggests that using bidirectional expression of eRNAs as a criterion for enhancer detection can inadvertently discard valid enhancers. As a concrete example, we performed enhancer detection on the THP-1 CAGE data set [13] by requiring bidirectional enhancer transcription using the previously suggested conditions [3] of an absolute directionality score <0.8, and bidirectional expression in at least one sample. Using these criteria, we detect 26 902 enhancers; if we do not require bidirectional transcription, we find an additional 101 910 enhancers (see Supplementary Methods for details). Of these, 19 928 (74.1%) and 62 621 (61.4%), respectively, overlap candidate *cis-*regulatory elements defined by ENCODE [23]. These results suggest that many valid enhancer candidates will be missed if balanced bidirectional expression is included in the criteria for enhancer detection.

## Discussion

Unidirectional and asymmetric expression of enhancers has occasionally been observed in eukaryotes [24–31]. While enhancer expression is indicative of enhancer activity [3, 32, 33], it remains unclear if enhancer transcription is due to transcriptional noise [34] or plays a biological role. For example, enhancer transcription may affect the local chromatin structure by displacing nucleosomes, may prevent gene silencing, or may stimulate the deposition of active chromatin marks [35]. Additionally, transcription factor binding to eRNAs may boost the local transcription factor concentration and hence their regulatory effect [36]. The direction of enhancer transcription would affect each of these mechanisms.

As some of the enhancers classified as having a consistent preferred direction of transcription may have a different preferred direction in cell types not included in FANTOM5, the number of switching enhancers obtained in this study is likely to be an underestimate. Detection of preferentially directional transcription may further be hampered by limitations in sequencing depth, leading to an overestimation of the number of bidirectionally expressed enhancers.

As previous evidence indicated bidirectional transcription of enhancers, balanced expression of eRNAs has been used as a criterion for enhancer detection from transcriptome data [3]. Our analysis shows that though an enhancer may be capable of transcription in either direction, in any given cell type transcription may be strongly biased toward one direction. Especially when analyzing deep-sequenced CAGE libraries obtained from one cell type, we advise against using balanced bidirectional expression as a requirement for enhancer detection, as it may inadvertently discard valid enhancer candidates.

## Supplementary Material

lqaf007_Supplemental_Files

## Data Availability

No new data were generated or analysed in support of this research.
