# Supplementary material for: Cell type-dependent directional transcription at enhancers
Source: NAR Genom Bioinform. 2025 Mar 7;7(1):lqaf007. doi: 10.1093/nargab/lqaf007 (PMC11886823; doi:10.1093/nargab/lqaf007)
Supplement: lqaf007_Supplemental_Files [file lqaf007_supplemental_files.zip › supplementary_materials.pdf]

Supplementary Methods

The downsampling analysis was performed by randomly selecting 4,948,682 CAGE tags, corresponding to the average number of CAGE tags in a FANTOM5 sample, without replacement from the 3,998,535,446 CAGE tags in FANTOM5 CAGE data pooled over samples. Of the selected CAGE tags, 22,740 mapped to enhancers. As only enhancers with at least 2 CAGE tags contribute to the estimation of the shape parameter α, we discarded enhancers that received only 1 CAGE tag, leaving 15,263 CAGE tags on included enhancers contributing to the estimation. This is slightly lower than the 19,639 CAGE tags found on average across FANTOM5 samples on enhancers with at least 2 CAGE tags (**Supplementary Table 1**).

Simulated data were generated by first calculating the distribution of CAGE expression levels across enhancers. This was done in two ways:

① To simulate the FANTOM5 pooled data, we calculated the number of CAGE tags of each enhancer summed across samples, and normalized to 1 to find the CAGE expression distribution across enhancers.

② To simulate the FANTOM5 data in a single sample, we calculated the number of CAGE tags of each enhancer in each sample separately. We then sorted the enhancers in each sample by their CAGE tag count in the sample, and normalized to 1 to find the CAGE expression distribution across enhancers in each sample separately. We then averaged this distribution to obtain the representative distribution of CAGE expression across enhancers in a single FANTOM5 sample.

Next, for each simulation we chose the total number of CAGE tags on enhancers, and used the multinomial distribution to assign this number of CAGE tags to enhancers using the distribution across enhancers calculated as described above (option ① to simulate pooled data, or option ② to simulate a single sample). For the estimation of the shape parameter α, we only included enhancers with 2 or more CAGE tags and discarded enhancers with 0 or 1 CAGE tags, as they do not contribute to the estimation of α. We counted the total number of CAGE tags on the included enhancers, and show this number on the horizontal axis in the panels of **Supplementary Figure 6**. For comparison, we indicated the total number of CAGE tags on included enhancers for the other data sets; these values are also listed in **Supplementary Table 1** in the column "Number of CAGE tags on included enhancers". Next, we estimated the value of the shape parameter α for each simulated data set, as well as its statistical significance using the likelihood ratio test. The results for option ① (simulating pooled data) are shown in **Supplementary Figure 6** panels A and B; the results for option ② (simulating a single sample) are shown in **Supplementary Figure 6** panels C and D.

To assess if variations in the directionality score of an enhancer across samples was statistically significant, we counted the number of forward and reverse CAGE tags for each sample with at least 10 CAGE tags in total, and performed the χ^2 test for each enhancer with at least 2 of such samples. If the χ^2 test gave a statistically significant (P-value < 0.05) result, we performed a binomial test with $p = 0.5$ on each of the samples separately. We concluded that the enhancer direction switched across samples if the binomial test yielded at least one sample in which the

number of forward reads was significantly (*P*-value < 0.05) greater than the number of reverse reads, and at least one sample in which the number of forward reads was significantly (*P*-value < 0.05) less than the number of reverse reads.

The directionality scores for the enhancers included in the reporter assay analysis were calculated using FANTOM5 Phase 1 libraries CNhs12325, CNhs12326, CNhs12327 for HeLa, and CNhs12328, CNhs12329, CNhs12330 for HepG2.

Detection of enhancers with balanced bidirectional expression was performed by using the scripts described by Andersson *et al.* (3, 4) using default parameters. Preferentially unidirectional enhancers were detected by taking the list of tag clusters generated by these enhancer prediction scripts, and discarding tag clusters overlapping a tag cluster on the opposite strand or a predicted bidirectional enhancer. We excluded bidirectional and unidirectional enhancer predictions located in promoters and exonic regions in hg38 by masking the 500 bp upstream and downstream regions of annotated TSSs, and as well as exons extended by 200 bp upstream and downstream, as described previously (37). We required all predicted enhancers to have at least 10 CAGE tags summed across samples.

Supplementary Figures

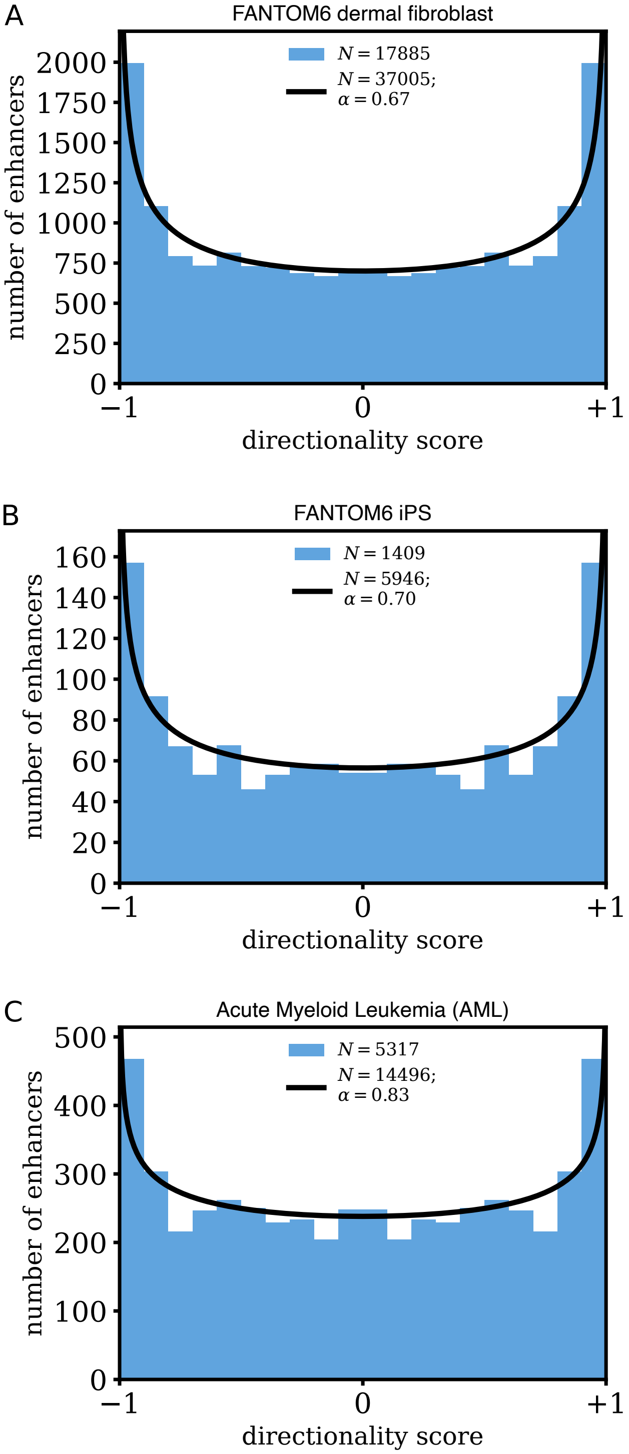

Supplementary Figure 1. Distribution of the directionality score in (A) FANTOM6 dermal fibroblast (14), (B) FANTOM6 iPS cells (15), and (C) AML primary cells (16). The estimated beta distribution is shown as a curve; the histogram was calculated from enhancers with at least 10 CAGE tags.

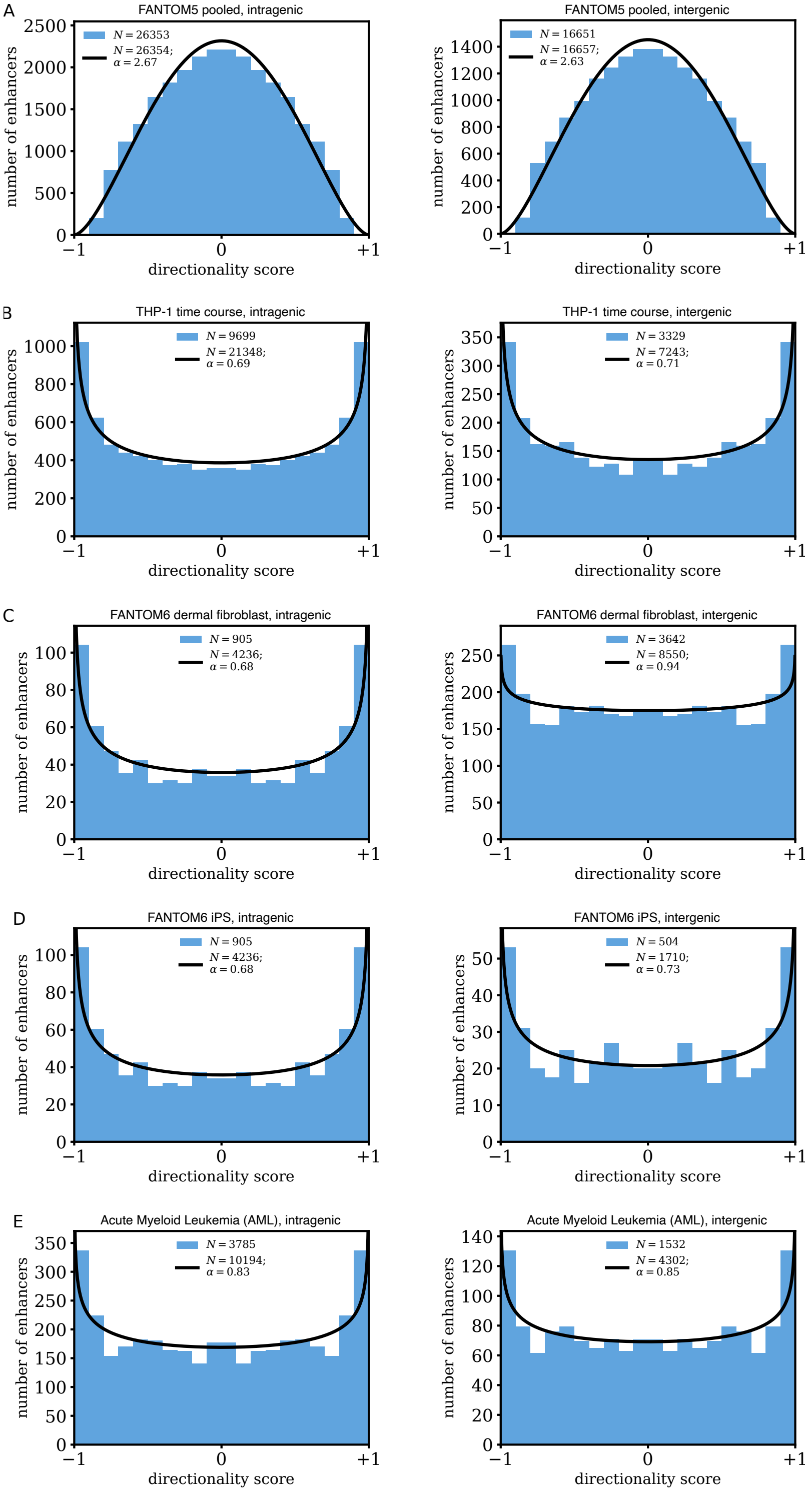

Supplementary Figure 2. Distribution of the directionality index in (A) FANTOM5 pooled data (17), (B) THP-1 (13), (C) FANTOM6 dermal fibroblast (14), (D) FANTOM6 iPS cells (15), and (E) AML primary cells (16), calculated separately for intragenic enhancers (left column) and intergenic enhancers (right column). The estimated beta distribution is shown as a curve; the histogram was calculated from enhancers with at least 10 CAGE tags.

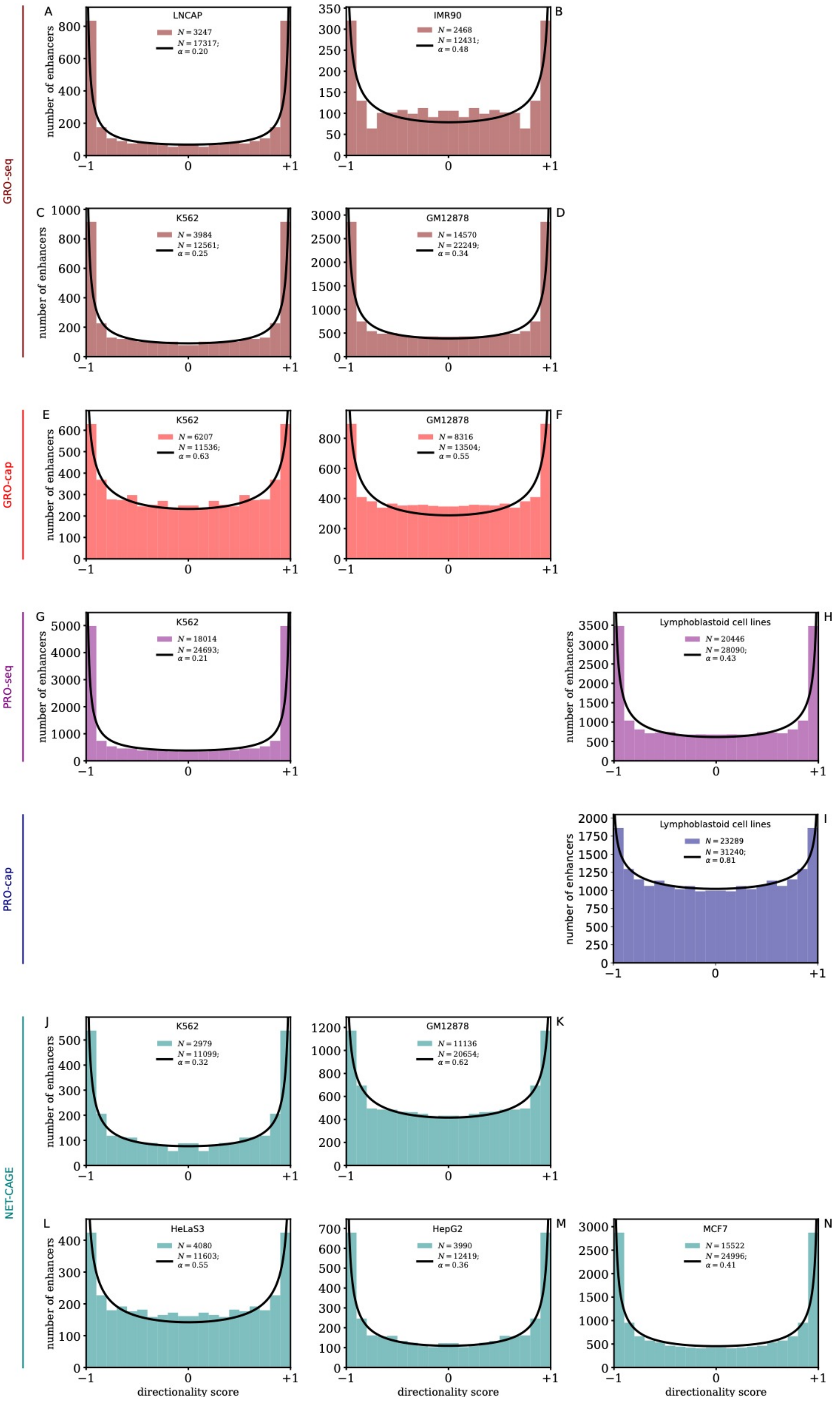

Supplementary Figure 3. Distribution of the directionality score in GRO-seq (panels A-D), GRO-cap (panels E, F), PRO-seq (panels G, H), PRO-cap (panel I), and NET-CAGE (panels J-N) data. The estimated beta distribution is shown as a curve; the histogram was calculated from enhancers with at least 10 tags. Cell lines were LNCaP (panel A) (38); IMR90 (panel B) (18); K562 (panels C, E, G (19) and panel J (20)); GM12878 (panels D, F (19); and panel K (20)); lymphoblastoid cell lines (panels H, I) (7); HeLaS3 (panel L) (20), HepG2 (panel M) (20), and MCF-7 (panel N) (20).

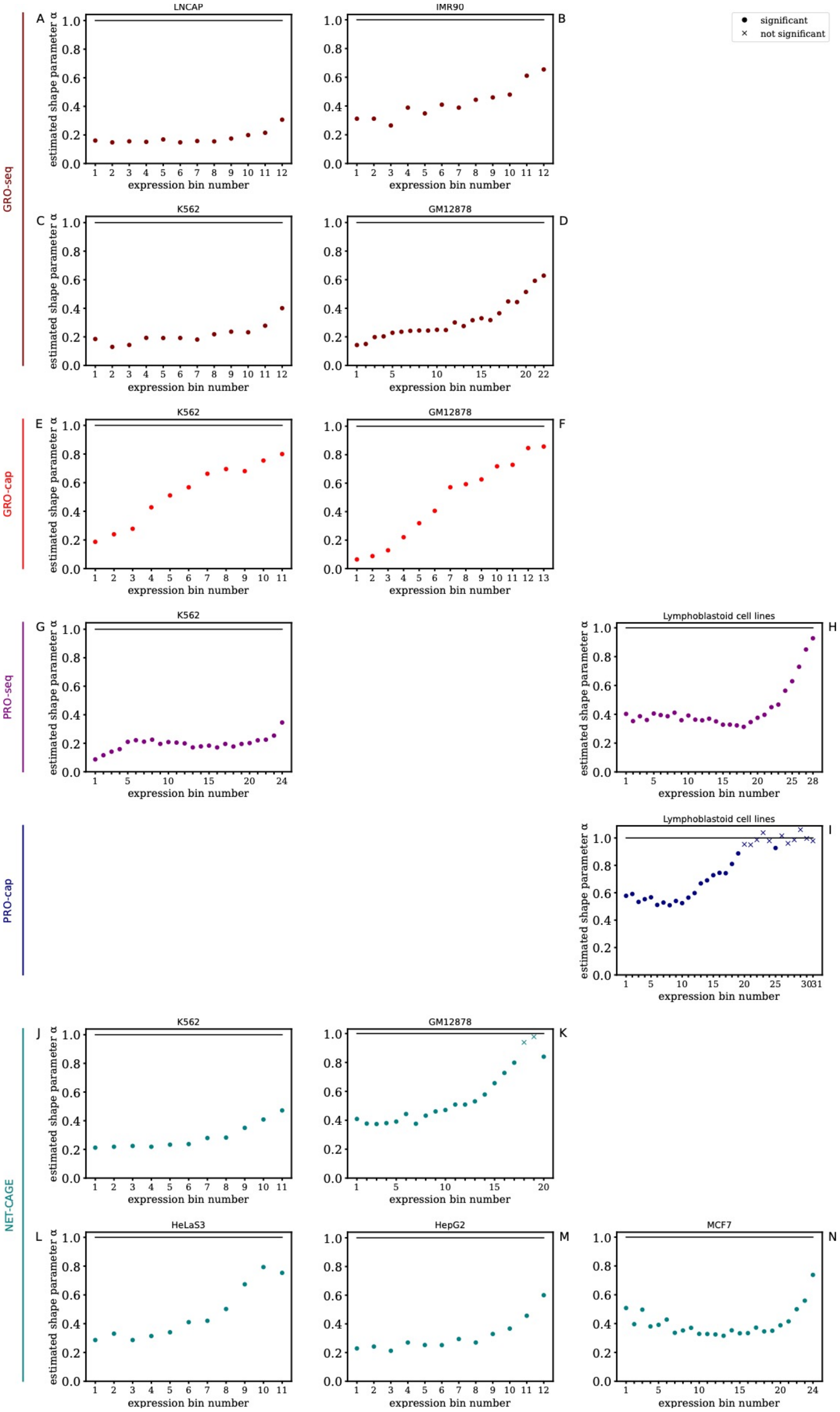

Supplementary Figure 4. Dependence of the directionality score on expression level in GRO-seq (panels A-D), GRO-cap (panels E, F), PRO-seq (panels G, H), PRO-cap (panel I), and NET-CAGE (panels J-N) data. The directionality score was calculated on bins of 1000 enhancers sorted by expression level. Cell lines were LNCaP (panel A) (38); IMR90 (panel B) (18); K562 (panels C, E, G (19) and panel J (20)); GM12878 (panels D, F (19); and panel K (20)); lymphoblastoid cell lines (panels H, I) (7); HeLaS3 (panel L) (20), HepG2 (panel M) (20), and MCF-7 (panel N) (20).

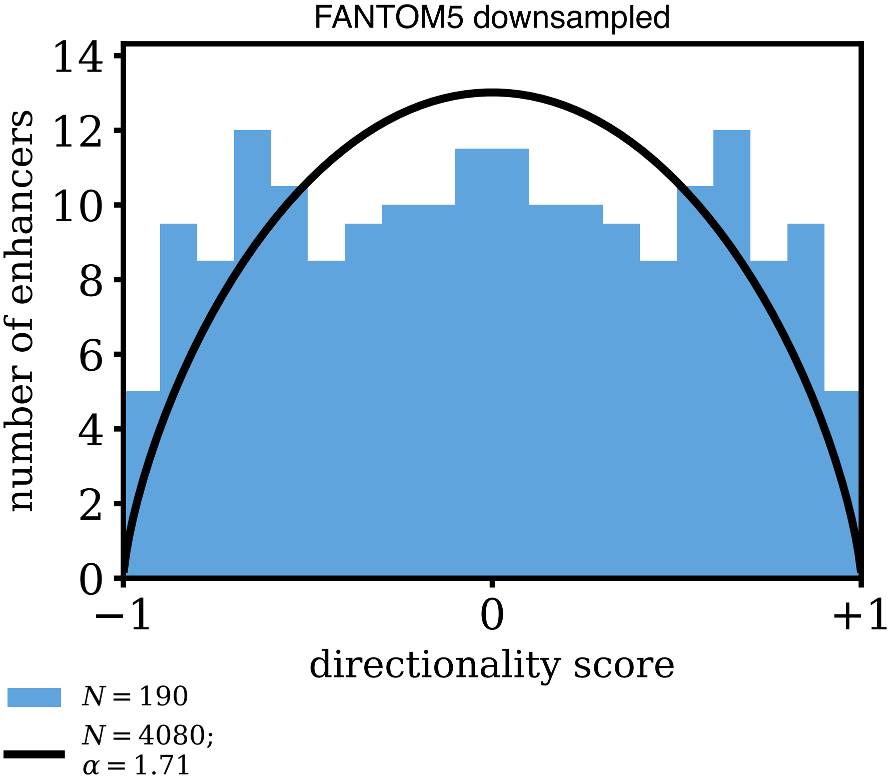

Supplementary Figure 5. Distribution of the directionality score in the FANTOM5 pooled CAGE data after downsampling to the mean size (4,948,682 CAGE tags) of one sample in FANTOM5.

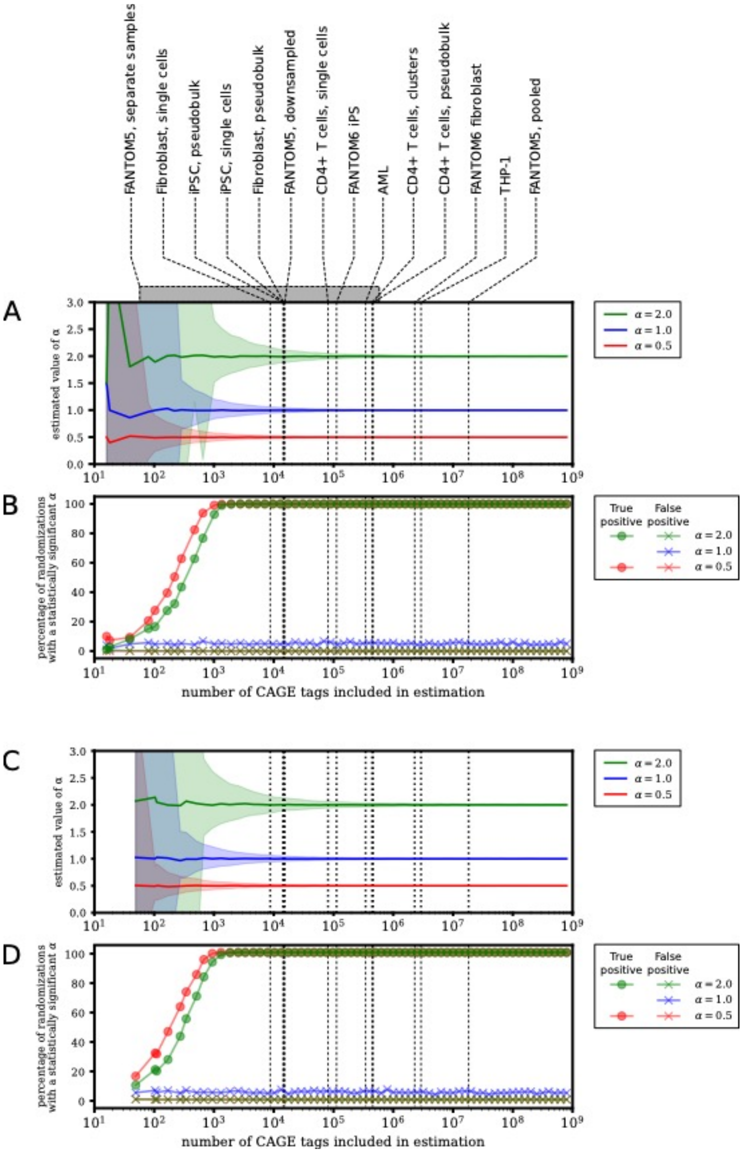

Supplementary Figure 6. (A, C) Estimated value of the directionality score for simulated data, as a function of the number of CAGE tags included in the estimation, for a concave distribution function with $\alpha = 2$, a uniform distribution ($\alpha = 1$), and a convex distribution function with $\alpha = 0.5$. The curve shows the mean value over 1000 simulations, and the shaded area shows the mean ± the standard deviation. (B, D) The percentage of randomizations for which the estimated directionality score was statistically significantly different from 1. At a sufficiently high number of CAGE tags, the estimated value of the directionality score for the concave or convex distribution was statistically significant for all randomizations. With a low number of CAGE tags, statistical significance could not be reached for the concave and convex distributions. Note that the false positive rate remained uniformly low independent of the number of CAGE tags, as shown by the blue curve ($\alpha = 1$). The dotted lines indicate the number of CAGE tags on included enhancers in each dataset (**Supplementary Table 1**). CAGE tags were distributed over the enhancers based on their expression data in the pooled FANTOM5 data (panels A, B) or by averaging over the FANTOM5 data separated by sample (panels C, D; see Methods for details).

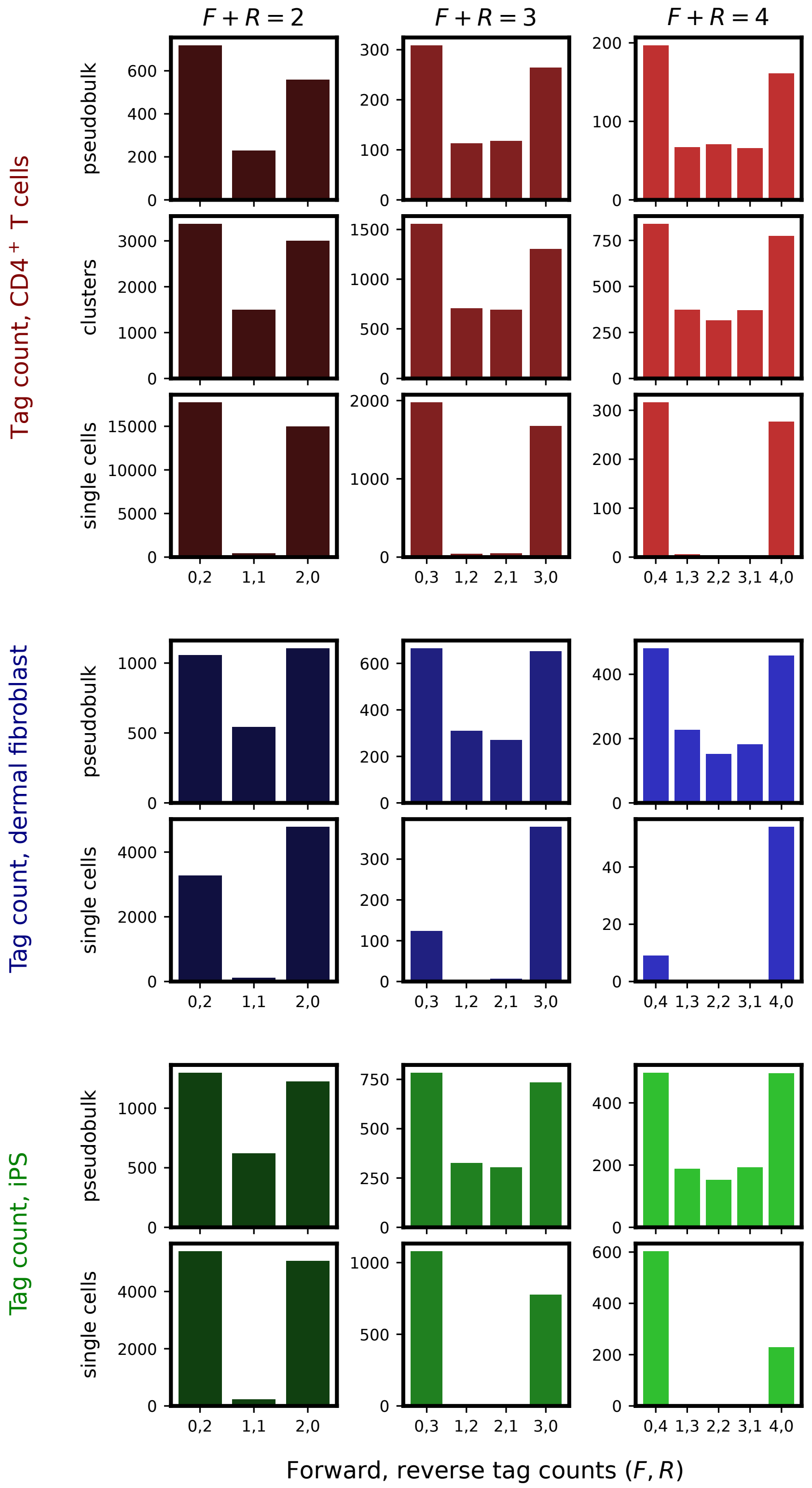

Supplementary Figure 7. Histograms showing the number of tag counts in CD$^+$ T cells, dermal fibroblasts, and iPS cells for all combinations of forward (*F*) and reverse (*R*) reads for all enhancers with $F + R = 2$, 3, or 4 tag counts, for single cells, clusters of CD$^+$ T cells, and pseudobulk data. In single cells, enhancer expression is almost exclusively unidirectional.

Supplementary Tables

Supplementary Table 1 [separately uploaded Excel file]. Number of CAGE tags and expressed enhancers in each data set. Included enhancers had at least two CAGE tags and therefore contributed to the estimation of the shape parameter α (see Methods for details). For the FANTOM5 separate samples, the minimum, maximum, and mean number across samples is shown.

Supplementary Table 2 [separately uploaded Excel file]. Directionality analysis of the FANTOM5 enhancers.

Supplementary References

37. De Hoon,M., Bonetti,A., Plessy,C., Ando,Y., Hon,C.-C., Ishizu,Y., Itoh,M., Kato,S., Lin,D., Maekawa,S., *et al.* (2022) Deep sequencing of short capped RNAs reveals novel families of noncoding RNAs. *Genome Res.*, **32**, 1727–1735.

38. Wang,D., Garcia-Bassets,I., Benner,C., Li,W., Su,X., Zhou,Y., Qiu,J., Liu,W., Kaikkonen,M.U., Ohgi,K.A., *et al.* (2011) Reprogramming transcription by distinct classes of enhancers functionally defined by eRNA. *Nature*, **474**, 390–394.
